
JOURNAL INFORMATION
==============================
NLM Title Abbreviation: ScientificWorldJournal
Journal ID: ScientificWorldJournal
NLM Journal ID: 101131163
Journal ID: TSWJ

The Scientific World Journal

ISSN: 2356-6140
EISSN: 1537-744X
Publisher: Wiley

ARTICLE INFORMATION
==============================
PMCID: PMC5901147
PMID: 29861695
DOI: 10.1100/tsw.2007.174
Article ID: 658969
Article version: 1
Subjects: Editorial

Editorial: Special Issue. Current Treatment of Localized Renal Cancer

Kirkali Ziya
Dokuz Eylul University, School of Medicine, Genito-Urinary Group, Inciralti, Ismir, Turkey
Publication date: 2007
Electronic publication date: 2007 Jun 22
Volume: 7
First page: 1046
Last page: 1046
Copyright: Copyright © 2007 Ziya Kirkali.
Copyright year: 2007
License: This is an open access article distributed under the Creative Commons Attribution License, which permits unrestricted use, distribution, and reproduction in any medium, provided the original work is properly cited.
License URL: https://creativecommons.org/licenses/by/3.0/

==============================
Editorial
Special Issue: Current Treatment of Localized Renal Cancer
*TheScientificWorldJOURNAL*, (2007) 7, 1046
TSW Urology
ISSN 1537-744X; DOI 10.1100/tsw.2007.174

TheScientificWorldJOURNAL
www.thescientificworld.com

### Editorial: Special Issue. Current Treatment of Localized Renal Cancer

Ziya Kirkali
*Dokuz Eylul University, School of Medicine, Genito-Urinary Group, Inciralti, Ismir, Turkey*

**E-mail:** ziya.kirkali@deu.edu.tr

*Published June 22, 2007*

---

Renal cell cancer (RCC) constitutes 3% of all solid neoplasms seen in adults and there has been an increase throughout the world. This increased incidence is mainly due to incidental detection by imaging for non-urological causes. Despite earlier diagnosis of smaller tumors, the mortality due to RCC is also on the rise. It appears that we need more effective treatment for this disease.

Both in retrospective large series and prospective trials approximately 15% of small solid renal masses are benign lesions. Dr. Volpe addresses the issue of the natural history of these small solid lesions and proposes that active surveillance may be an option at least in some elderly or unfit patients.

Surgery remains to be the only effective therapy for RCC. For many years, radical nephrectomy has been the standard treatment for localized disease. Due to more frequently seen smaller tumors organ and nephron-sparing approaches have gained wide acceptance by the urologists and the patients. Vander Eeckt and colleagues discuss the role of open surgery for localized RCC. While open radical nephrectomy still has a place in some large and invasive tumors, most small tumors today can be safely treated by open nephron-sparing surgery.

RCC is a disease where laparoscopic and minimally invasive therapies have been widely investigated. Dr. Eskicorapci and colleagues discuss the role of laparoscopy in the treatment of localized RCC. In most cases, it has been the new standard of care with less morbidity and quick recovery providing similar oncological outcomes. Laparoscopy will probably be used more frequently in the future for nephron-sparing procedures.

Dr. Lovisolo and colleagues report on the advances in the use of minimally invasive ablative therapies in localized RCC. They discuss the role of laparoscopic and percutaneous cryotherapy, radiofrequency ablation, laser thermal ablation and high intensity focused ultrasound in the treatment of RCC. Although some of these ablative modalities can be used for selected individuals with small renal masses, we need more evidence on their efficacy and safety before embarking on a more widespread use.

I would like to thank the authors for their excellent contributions to this Special Issue of TSWJ on Localized Renal Cell Cancer, and I hope this issue will be useful to the practicing urologists who treat this disease.

---

**This article should be cited as follows:**

Kirkali, Z. (2007) Editorial: Special Issue. Current treatment of localized renal cancer. *TSW Urology* 2, 151. DOI 10.1100/tswurol.2007.130.

---

©2007 with author.
Published by TheScientificWorld: www.thescientificworld.com
